# What Are the Main Drivers of Young Consumers Purchasing Traditional Food Products? European Field Research

**DOI:** 10.3390/foods7020022

**Published:** 2018-02-12

**Authors:** George Vlontzos, Leonidas Kyrgiakos, Marie Noelle Duquenne

**Affiliations:** 1 Department of Agriculture, Crop Production and Rural Development, University of Thessaly, Volos 38446, Greece; kyrgiako@agr.uth.gr; 2 Department of Planning and Regional Development, University of Thessaly, Pedion Areos, Volos 38333, Greece; mdyken@uth.com

**Keywords:** traditional, local, consumer behavior, Principal Component Analysis

## Abstract

In this research, the attitude of European young adults (age 18 to 30 years) regarding their consumption of local and traditional products was examined. The survey was conducted on a sample of 836 consumers from seven European countries (Greece, Bulgaria, Romania, Slovenia, Croatia, Denmark and France). Data collection was made by distributing a developed questionnaire through social media and university mail services. Principal Component Analysis (PCA) was used to identify consumer perception comparing the overall sample with two subsets (consumers from Eastern and Western European countries). Six major factors were revealed: consumer behavior, uncertainty about health issues, cost, influence of media and friends and availability in store. Young adults had a positive attitude to local and traditional food products, but they expressed insecurity about health issues. Cost factor had less of an influence on interviewees from Eastern European countries than those from the overall sample (3rd and 5th factor accordingly). Influence of close environment was a different factor in Eastern countries compared to Western ones, for which it was common to see an influence from media. Females and older people (25–30 years old) have fewer doubts about Traditional Food Products, while media have a high influence on consumers’ decisions. The aim of this survey was to identify the consumer profiles of young adults and create different promotion strategies of local and traditional products among the two groups of countries.

## 1. Introduction

In 1919, French agronomists persistently requested their government to protect by law the quality of their wines from the Bordeaux region [1] and set the basis for the Geographical Indications (GIs) legal framework of the European Union (EU). Since 14 July 1992, the EU has established and implemented a protective agenda for products with locality characteristics, regulating (R2081/92 and R2082/92) called the Protected Designation of Origin (PDO), Protected Geographical Indication (PGI), as well as Traditional Specialities Guaranteed (TSG) for food products. Moreover, the European Union’s (EU) bilateral agreements with Canada, USA and China provide the ability to promote the aforementioned products under the European legal framework, confirming in practice that locality characteristics can be an economic growth factor for these specific production areas [2]. Later on, this legal approach was accepted and adopted by non-EU countries as part of bilateral agreements signed between them. Such a case is the bilateral agreement between the EU and Japan with the Japanese government to accept the GIs characteristics for more than 200 European Geographical Indication products, thus proving the dynamics of such production methods even in regions with completely different culture. Additionally, Japan has established a similar legal framework for protecting locally produced agricultural products, thus adopting the European rationale [3]. The number of submitted geographical indication products is increasing in number, as are their sales, indicating that there is a growing universal interest in products with certain sensory characteristics (e.g., South Korea) with a unique identity of production process accompanied by a tradition as to how and when people taste them [4]. Until today, 1403 products have been submitted to the EU as PDOs, PGIs and TSGs, with the majority of them belonging to Southern European countries (Italy (293), France (244), Spain (194), Portugal (138), and Greece (105)). Only a few of those products originate from Northern European countries (Finland (10), Sweden (8), Denmark (6)) [5].

In an attempt to provide a definition of Traditional Food Products (TFPs) for European consumers, Vanhonacker et al., underline the importance of cultural and territorial identity, transferability from one generation to another and processing and sensory characteristics and describe different attitudes between countries [6]. The TFPs concept has been analyzed, evaluated and tested through personal interviews, resulting in the same definition as mentioned above [7]. In another survey about locality index, consumers expressed their positive feelings both for consuming local products and creating value for the local community, but there were also negative perspectives for cost and difficulties to find them in stores [8]. The EU’s definition of TFPs introduces the sustainability term referring to local environments and underlines the importance of labeling [9]. Additionally, the Euro Food Information Resource (FIR) consortium consider TFPs products to consist of specific raw materials and be produced under predefined production processes, emphasizing locality characteristics, in order to maintain tradition [10].

Up to now, a literature review describes an overall image of the European population’s attitude towards TFPs. The key points are the following:

- Adolescents with a higher education level are considered as a health-sensitive group, while at the same time consumption of TFPs is considered as a healthy attitude too [11].
- Young adults present a more snack-related food behavior compared to the overall population. This attitude is an obstacle for TFPs purchase [12].
- Even from a human physiology aspect, young people receive more sensory characteristics than older ones [13]. This tendency hints at an increase of TFPs usage.

Among others, widely accepted behavioral models are the Theory of Planned Behavior (TPB) and the Health Belief Model (HBM). In this research, a combination of these is used to clarify attitudes connected with traditional food consumption, and the perceived risk associated with such attitudes for young adults’ health. The final goal is to determine a marketing strategy that fulfills young consumer needs.

The Theory of Planned Behavior (TPB) [12,13] is an extension of the Theory of Reasoned Action (TRA) that was developed in 1980 by, Ajzen and Fishbein [14,15,16]. TPB examines attitudes on three levels: (a) Attitude towards the behavior; (b) Subjective norms and (c) Perceived behavioral control.

- (a) Attitude towards behavior is the degree of approval or disapproval of certain behavior by a person. Referring to the literature, the rising interest for products with unique characteristics combined with an increasing consumption rate has created a positive image of TFP [17]. However, there is a negative attitude regarding health issues, and production and manufacturing procedures being implemented during processing, especially from people with high education standards [18].
- (b) Subjective norm is the social attitude about a person’s behavior. (b) Norms derive from close environment (family, friends) and media. Family is responsible for creating a person’s food and eco-friendly attitudes [19,20], while information from a friend’s mouth can substantially influence a person’s behavior [21]. There is evidence that the increase of cooking is not a random phenomenon, with the industry aiming to further support and promote this tendency to thus increase consumption of TFPs [22]. Additionally, inside the EU, there appear to be many dietary differences, mainly based on different cultures. These can be considered as a barrier for consuming certain products in different regions [23]. TFPs can be a sign of identity and a link to their roots for people that live away from their home countries, differentiating them from inhabitants [24].
- (c) Perceived behavioral control is the level of difficulty for a person to perform a certain behavior. Safety standards, longer self-life and consumer-friendly appearance require traditional food products to be modernized without abolishing their identity. Innovation in TFPs introduces the adoption of new technologies during the production process. Nutritional value and packaging must comply with safety issues related to national or European legislation. Quality maintenance is crucial for consumers’ acceptance (or not) of an innovative intervention in TFPs. New technologies can help certain TFPs to be accepted by elderly consumers (e.g., low fat, less salt) [25]. Additionally, innovation is indispensable for TFPs, in order for them to be accepted by food supply chain protocols being implemented mainly by retail chain stores. TRUEFOOD Integrated project (2006–2010),financed by the EC, communicates a series of promotion tools for TFPs, based on modern surveys which assess the current market needs [26]. These determining factors shape the intention of a person to perform a behavior. Strong intensions are more likely to be expressed as behavior than weak ones.

The Health Belief Model (HBM) is being used in the food sector in order to measure individuals’ perception of certain products and their opinions about effects on their health [27]. Socioeconomic characteristics like age, gender, ethnicity, level of education, income and type of employment, are also estimated in this model. According to Pechey et al., 60% of people with low socioeconomic status buy food with a hazardous effect on their health [28]. Men are found to be more spontaneous and enthusiastic while women focus on morals when purchasing a certain product [29]. Southern Europe presents a higher consumption level of TFPs than the North, meaning that ethnicity does the affect relationship with TFPs [30]. Pieniak et al., in their study among six European countries, identified difficulties in the consumption of TFPs, as well as strong doubts regarding health issues [31]. There is, however, a fairly important issue regarding the etymology of the word *Ethnicity* for food consumption, because it influences the performed behavior of a person not only on a national level but also on a regional one [32]. In the same survey, men were found to spend more on organic food than women.

Cost can be a significant factor on final purchasing decisions, as there are groups of people that are described as hyper-sensitive, especially on online markets [33]. Sales of healthy products increased by 10%, when they were on discount, and educational level was not verified to be a significant factor [34]. Cost was proved to be in close relation with quality, as it was expected to be. In survey of Di Vita et al., the assessment of consumer purchasing decisions on different types of olive oil (conventional, PDO and organic) showed that the highest influence factor was price for people from rural areas but, for urban populations, the area of origin was the most important one [35]. In another survey for purchasing local jams, quality had a major impact [36]. Moreover, cost, availability and preparation time are limiting factors for TFPs consumption [37]. From the consumers’ viewpoint, organic products are considered high quality and more expensive than conventional products. On the other hand, TFPs preserve quality elements but they are not accompanied with high price anticipations [38]. Finally, Willingness to Pay (WTP) was investigated for TFPs because they are considered as premium products and thus they should have a different pricing policy [39].

Experience in store should be positive, pleasant, and reliant as the survey of Walsh et al. indicates [40]. Trust between supplier and seller, strength of habit and personal preferences are the most significant factors for meat purchasing behavior [41]. The same issue of trust about the local market is confirmed by Migliore et al., who also mention health issues as barriers for TFPs consumption [42]. Pleasure rates are higher when people are informed about the products that they are going to taste, especially when this information is received by young consumers [43]. Additionally, there are cases where local people are habitually connected with traditional products, making their consumption a necessity for them [44]. Positive images of using TFPs from celebrations and special occasions accompany them in their adulthood [45].There is also a rising interest of people caring not only for quality, but also for animal welfare [46]. Small and medium enterprises (SMEs) should take advantage of this face to face communication with local people and promote themselves [47]. Finally, the special identity of TFPs characterizes them as unfavorable for new markets and they should be promoted appropriately to gain entrance, as well as market share [48].

The main aim of this study is to investigate young consumers’ motives for TFPs consumption, by implementing the Theory of Planned Behavior and the Health Belief Model. This survey took place in seven European countries (Greece, Bulgaria, Romania, Slovenia, Croatia, Denmark and France) only with young adults, so as to clarify the rationale leading to specific attitudes for TFPs consumption.

## 2. Materials and Methods

A literature review provided the essential information for the identification of the most important factors that influence the European consumers’ perceptions about local and traditional products. More specifically, social, demographic and psychological factors were of major importance in influencing the decision-making process on TFPs consumption. The EU appears to have a high heterogeneity when it comes to cultural aspects, which makes it an ideal area for such surveys [49]. In addition, shop selection criteria and purchasing attitudes have as a driving force a more environmentally friendly consumer profile. They have been included in this survey in order to quantify their impact. As mentioned above, there is a general knowledge about European perceptions on TFPs but there is a need to broaden the knowledge about consumer behavior of the young consumer community, which is in fact the dynamic purchasing market for the next 30years [39,40].

For this reason, questionnaire-based field research was conducted at a European level, during the period September–October 2016 (See the Supplementary Materials). An overall sample of 836 respondents was created for the purpose of this study.

The questionnaire was subdivided into three subsections. In the first part of the questionnaire there were questions about the social and demographic data of the respondents, such as gender, age, income and level of education. The second part introduced to consumers a group of proposals. More specifically, this part consists of four subgroups of questions examining perceptions about TFPs: consumer behavior (subgroup 1), health issues (subgroup 2), cost (subgroup 3) and degree of influence from different factors (subgroup 4). The third part focused on re-examining the respondents’ opinions about the previous referred groups and also there were 2 questions referring to the availability of products in stores (group 5). For these questions, a Likert scale was used from 1 (Absolutely disagree)–5 (Totally agree).

The questionnaire was initially tested on a smaller sample of respondents before the main distribution in eight (8) European countries (Greece, Denmark, England, France, Slovenia, Croatia, Bulgaria, and Romania) to verify that the questions were adequately written and understandable. This test took place in Greece and Denmark, as they represent Eastern and Western European countries. Various methods were used for distributing this questionnaire. These were mailing lists, social media, like Facebook and Instagram, as well as personal interviews.

In order to identify the drivers for TFPs consumption, a Factor Analysis (FA) was performed to determine whether the data relative to consumers’ attitudes can detect different categories of consumers. The major objective of the Exploratory Factor Analysis (EFA) is to explore the formation of a set of interrelated variables without imposing any fixed structure of the outcome. The extraction method was Principal Component Analysis (PCA), based on Varimax rotation.

For the reliability of results, two tests were used:

- The Kaiser-Meyer-Olkin index (KMO);
- Barlett’s spherisity test.

The Kaiser-Meyer-Olkin index (KMO) explores the suitability of the sample to be analyzed. In particular, it examines the relative magnitude of correlation coefficients in relation to the partial correlation coefficients. The higher the correlation, the better the analysis of the sample. When the KMO value is too low, less than 0.5, this means that the factorial analysis will not have satisfactory results. Values above 0.7 are acceptable for analysis, and 0.8 are considered as very good.

let R = (rij) (p × p).(1)

where R is the correlation matrix, rij is the partial correlation, and p is the number of variables.

With Barlett’s spherisity test, it is examined whether the observed correlation of the data table, differs statistically from its actual identity. Therefore, the zero hypothesis (H_0_) is that the data is arranged in a rectangular form. This assumption is controlled at a 5% materiality level

PCA was developed by Charles Spearman. The acceptance of this methodology is wide in areas such as psychology, market research, the labor market and human resource management, where quantitative measurements produce results that help to make critical decisions [50,51]. It is a method that is being used to analyze large datasets, and it eliminates information in order to form groups (components). Two main elements of PCA are: factor loading and sum of squared loadings. Factor loading interprets whether a set of data affects the factor that has been formed. If the participation ratio is fairly low, below 0.4, then it is assumed that its influence is too small and for this reason it is rejected. When the loadings vary between 0.5–0.7, the degree of participation is considered satisfactory and from 0.7 and above, the factor is very well supported. The sum of squared loadings describes the amount of variance of a factor. PCA was performed with IBM SPSS Statistics for Windows, Version 23.0, IBM Corp., Armonk, NY, USA.

## 3. Results

### 3.1. Demographics

The sample consisted of 295 males and 541 females with a mean age 23.3 (Standard Deviation = 3.34). A higher rate for female respondents has been observed by other papers as well [52,53,54,55,56]. Educational level is fairly high with 57.68% of participants already holding a degree. Low income was another characteristic of this dataset with 58.49% receiving a monthly income lower than 500 € per month. Unemployment ratio was a little lower than EUROSTAT (Statistical office of the European Union) (7.7%) for 2016 [57] compared to the overall sample (5.98%). The questionnaire was based on the above-mentioned Theory of Planned Behavior (TPB) and Health Belief Model (HBM).Table 1 gives an overview of overall sample characteristics (*n* = 836), which was derived from questionnaires completed by 18–30 year old people from different European countries (Greece, Bulgaria, Romania, Slovenia, Croatia, Denmark, France, and England). About 70% of the participants are aged 18 to 24, while about 30% are 24–30. This is mainly due to the way the questionnaires were distributed, because distribution took place to a large extent through university networks. That explains the lower unemployment rates of the sample, compared with those of the EU. Moreover, educational level of the sample is rather high, with 35% holding a postgraduate degree. In terms of income criteria, about 60% have an income of <500 €/month, while 22% are in the income class of 500–1000 €/month, covering a total of 82% of the distribution.

Further analysis was inducted, dividing the overall sample in two subgroups; Subset 1 referring to Eastern European Countries (Greece, Bulgaria, Romania, Slovenia, Croatia) and Subset 2 referring to Western ones (Denmark, France, England) in order to further investigate possible significant differences. For this reason, Table 2 presents an overview of the sample characteristics resulting from Subset 1 questionnaires (*n* = 569), which consist of 34.6% of men and 65.4% of women. About 75% of participants were aged 18–24 while about 25% were aged 24–30. The educational level of the sample is quite high since 32% of them hold a master's degree, while about 75% have incomes <500 €/month, and 14% have income of 500–1000 €/month.

Sample characteristics from Subset 2 (*n* = 267) are presented in Table 3, which consists of 36.7% of men and 63.3% of women. About 60% of participants are aged 18–24 while about 40% are aged 24–30. The educational level of the sample is quite high, with 41% holding a postgraduate degree. There are noticeable differences with respect to Table 2 concerning income criteria, since approximately 22% have an income of <500 €/month, while 37% are in the income class of 500–1000 €/month, covering a total of 82% distribution.

To sum up, it is noted that the overall sample refers to young people with an average age of 23 years, and a high educational level, while the economic criteria differ for the individual categories, with Western countries having higher monthly incomes.

### 3.2. Principal Component Analysis Results

#### 3.2.1. Overall Sample

For PCA of the overall sample (*n* = 836), KMO index (=0.793) showed a high degree of consistency between variables. Analysis carried out revealed five main factors reflecting 61% of total variance. Table 4 presents the factor loadings, which are quite satisfactory (>0.500) to very satisfactory (>0.700), except for two cases where the index value is marginal (0.400–0.500). (Q2.4 *People, whose opinion is important to me, approve of buying and using local and traditional products* (0.470), Q3.12 *My friends influence me to consume local and traditional products* (0.453))

Moreover, four questions were removed due to their very low loadings (<0.200)

Q2.6 *It depends on me if I will consume local and traditional products or not*;

Q2.7 *I don’t feel good when other people see me buying local and traditional products*;

Q3.1 *Health is better than wealth*;

Q3.8 *I buy local and traditional food products from small local shops*.

#### 3.2.2. Eastern European Countries

For Eastern European Countries (*n* = 569), KMO index (=0.783) showed a very good degree of consistency between variables. Analysis carried out revealed six main components reflecting 68% of total variance.

#### 3.2.3. Western European Countries

For the Western European sample (*n* = 267), the KMO index (=0.770) and components reflective (57%) of total variance, yielded fewer results compared to the previous sets. This is mainly due to the small number of questionnaires completed. However, the ratio between the number of observations and the number of variables is quite strong (267/16, approximately 17 per variable, when, in the literature, 10 observations per variable is considered as a very satisfactory ratio).

Finally, by comparing the importance of the components and their rating according to socio-economic characteristics (gender, age, education and income), some differences can be found, which are summarized in the following table.

## 4. Discussion

### 4.1. Overall Sample

1st Component: Consuming TFPs is a good and healthy consumer behavior. In this component, it appears that women tend to give even greater importance to hygienic behavior (*p*-value < 0.05), confirming that there are differences in gender consumption behavior, as described in the literature review.

2nd Component: Emphasis is given on questions about food safety issues of TFPs. It is worth noting that this feeling is negatively correlated with age. In addition, the higher the level of education, the fewer safety issues appear.

The first two components refer to two different perceptions but the first component (positive perception) is almost two times more important than the second one (negative perception). As in the bibliographic review, it is emphasized that consumers are rather skeptical about their use due to the lack of food safety criteria that are being observed during the production process.

3rd Component: The role of the economic parameter does not overshadow the first component, which highlights positive factors such as Healthy behavior. This is perhaps due to the fact that, even if local products are sometimes more expensive than conventional ones, they provide consumers with an additional degree of satisfaction. This additional satisfaction, in comparison with conventional products, provides hints for extra loyalty between producers and consumers of TFPs.

4th Component: Explains the influence of the media and the close environment. The role of media and friends increases with age (positive correlation). On average, influence is more important for women than men.

5th Component: The last component is the degree of availability of local traditional products. Consumers do not have a particular problem with finding local products, since both variables have a high degree of participation (>0.700).

### 4.2. Eastern European Countries

The main difference with respect to the overall sample is the financial benefit resulting from the consumption of local traditional products. For young people in Eastern countries, this parameter does not appear very important: it is now the 5th component in order of importance versus 3rd in the 1st model.

It is worth noting that the opinion of close environment is in this case an important and distinct component (4th Component) as opposed to the young people of Western European countries. People of Eastern European countries seem to have closer relations and thus are capable of influencing each other more than people of Western European societies.

Table 5 presents the six (6) different components for local and traditional products, with the main component recommending that the purchase and consumption of local and traditional products is good consumer behavior. The second component refers to food safety issues, while the 3rd and 4th components appear to be the influence of the media and the environment of close friends. Although media seem to affect consumers, the importance of close environment, which is not a separate factor for Western European countries, is underlined. As already mentioned, the 5th Component expresses the economic factor, while the 6th component expresses consumers’ inability to locate local and traditional products.

### 4.3. Western European Countries

Table 6 presents the results for the Western European countries. The first component is particularly strong, consisting of 9 variables. Healthy behavior is directly related to the opinion of close environment as well as financial benefit, which is not a separate parameter. As a consequence, the economic aspect for young people in the Western world is not as important as the previous group and is not, by itself, a dominant factor. The second component refers to the impact of food safety issues with a large contribution of individual factors (>0.750), which once again confirms the lack of trust for TFPs. The third component expresses the degree of influence from the media, which appears to be a component in itself. Finally, in the fourth component there is an availability issue, as many declare that it is difficult to find them, while others verify that traditional products are available in supermarkets.

It is worth mentioning that the relationship of the components being found for both Eastern and Western European countries with the socio-economic characteristics of the sample, provide new information about the impact of them on TFPs consumption. More specifically, Table 7 illustrates these findings. In Eastern European countries the gender, age and education parameters are positively related with the healthy behavior aspect of TFPs consumption. Additionally, there is a positive relationship among all the socio-economic parameters with the role of media, but only the income parameter is related with the impact of the close environment and the financial benefits being gained by the consumption of TFPs. As regards the Western European countries, the gender and the income parameters are significantly related with the healthy behavior aspect. The food safety issue is related with the age and the income characteristics of the sample. Finally, the availability component is significantly related with the income parameter.

Given the findings of this survey, it is worth clarifying promotion methods for each market segment. In particular, two clear consumer profiles are presented in Table 8. Five main components can be considered as significant pillars for introducing and implementing a promotion plan for local and traditional products.

1st Component: It is very positive that the consumption of local and traditional products is considered as good consumer behavior for young consumers in both Eastern and Western European countries. Therefore, since the perception of these products is already positive, there is no need to further insist on this issue.

2nd Component: TFPs should feel safe to consumers both in terms of production process and packaging. The safety issue is extremely important. Almost all research findings verify this, with this particular field research confirming it as well. It is therefore of major importance that TFPs should have all corresponding certifications in order to eliminate safety concerns from consumers. The same sense of safety should be depicted on packaging, by using the appropriate technology and aesthetics.

3rd Component: The Cost parameter was one of the factors introduced in the questionnaire. But it was quite ambiguous how it affects consumer behavior and, more precisely, younger consumers with lower incomes. Therefore, it appeared that cost is the 5th Component for the Eastern countries compared with the overall sample where it appears as the 3rd component. This was quite surprising, as eastern European countries, facing greater financial difficulties, seem to be willing to pay more for purchasing quality products and, more specifically, TFPs. Therefore, for firms producing or marketing such products, there is a potential for greater profitability in Eastern European countries.

4th Component: According to the Theory of Planned behavior, individuals appear to be affected by subjective norms. It seems that the opinion of close environment is, for the sample of the Eastern European countries, a fourth component in contrast to the young people of Western European countries, which, in that case, is not a separate component. This information can be immediately utilized as it is fully understood that young people in Eastern countries are more easily affected by their friends or closely related people. In addition, thematic events could be organized referring to such products and their uses so as to give an overall approach of their special characteristics to the public. In this way, they have the potential to influence both participants and individuals from their nearby environment, influencing them positively about their products.

5th Component: Availability, which is a separate component for all three calculations, appears to be of great significance. It is worth mentioning that interpretation of results is a model of analysis of perceptions rather than events. In other words, consumers believe that these products are not in hypermarkets even if they are there. This is a fairly serious problem, because, if consumers have difficulty finding them, then they are not able to buy and consume them. For this reason, it is proposed either to place TFPs in separate places in supermarkets, or for companies distributing their TFPs through hypermarkets, to create special stands for their products under a clear thematic approach, so as to be easily recognizable by consumers. It could also be part of an advertising campaign, focusing, among other issues, on the fact that these products can be found in specific chain stores.

## 5. Conclusions

This study focuses on the consumers’ attitudes and perceptions of TFPs, trying to describe the factors driving their final consumption choices. Through this research, the profile of new consumers, who will be the most important market segment of the next decades, is described. The main research findings are summarized as follows:

- Positive image for TFPs among young adults;
- Safety issues are highly important and thus TFPs should verify safety by introducing the appropriate certifications and packaging;
- Cost is a factor that affects less young adults from Eastern European countries more than those from Western countries;
- Media affect more adolescents from Western countries while influence from close environment is more significant for people from Eastern European countries;
- Availability of TFPs is an issue for young consumers and certain actions from suppliers should be taken towards eliminating this issue.

These findings verify to a large extent previous ones obtained by similar surveys that are presented in the literature review section. It is essential to undertake further research by focusing on both specific markets and products. As already mentioned, Europe is an area with many different cultures and each case should be examined separately. This new knowledge is essential for formulating more efficient promotion strategies towards increasing the market share of TFPs globally, by incorporating the values of European culture into dietary habits. Additionally, the implementation of widely accepted behavioral models, adjusted to the specific characteristics of this research, led to secure and verified findings. The verified nutritional value of TFPs is not perceived in a totally successful way by young consumers, despite the fact that various promotion strategies have been implemented for this cause. This study proves that there is a need for more focused promotion strategies, avoiding horizontal approaches, thus increasing the effectiveness and efficiency of such interventions.

## Figures and Tables

**Table 1:** Demographics of overall sample (*n* = 836).

	Male	Female	Summary
Gender	295	541	836
(%)	35.29	64.71	
Age (Average)	23.70	23.10	23.30
Standard Deviation	3.58	3.22	3.34
18–24(%)	24	48	72
24–30(%)	11	17	28
Education level (%)			
High school graduate	42.71	42.14	42.34
Bachelor	34.24	35.49	35.05
Master	18.98	19.59	19.38
PhD	4.07	2.77	3.23
Income month (%)			
<500 €	75.57	22.1	58.49
500–1000 €	14.94	37.08	22.01
1000–1500 €	6.50	16.85	9.81
1500–2000 €	1.05	6.74	2.87
2000–2500 €	0.88	6.74	2.75
2500–3000 €	0.18	1.87	0.72
>3000 €	0.88	8.61	3.35
Job Status (%)			
Employed	24.41	20.70	22.01
Unemployed	5.42	6.28	5.98
University Student	70.17	73.01	72.01

**Table 2:** Subset 1 sample (*n* = 569).

East European Countries			
	Male	Female	Summary
Gender	197	372	569
(%)	(34.0)	(65.4)	
Age (Average)	23.3	22.7	22.9
Standard Deviation	3.64	3.18	3.36
18–24(%)	25	52	76
24–30(%)	10	14	24
Education level (%)			
High school graduate	49.75	48.66	49.03
Bachelor	32.49	31.99	32.16
Master	12.69	15.59	14.59
PhD	5.08	3.76	4.22
Income month (%)			
<500 €	70.05	78.49	75.57
500–1000 €	14.72	15.05	14.94
1000–1500 €	10.15	4.57	6.50
1500–2000 €	1.02	1.08	1.05
2000–2500 €	2.03	0.27	0.88
2500–3000 €	0	0.27	0.18
>3000 €	2.03	0.27	0.88
Job Status (%)			
Employed	17.77	16.40	16.87
Unemployed	5.08	5.65	5.45
University Student	77.16	77.96	77.68

**Table 3:** Subset 2 sample (*n* = 267).

West European Countries			
	Male	Female	Summary
Gender	98	169	267
(%)	36.70	63.30	
Age (Average)	24.5	23.7	24.0
Standard Deviation	3.3	3.2	3.2
18–24 (%)	22	39.7	62
24–30 (%)	14.7	23.6	38
Education level (%)			
High school graduate	28.57	27.81	28.09
Bachelor	37.7	43.20	41.20
Master	31.63	28.40	29.59
PhD	2.04	0.59	1.12
Income month (%)			
<500 €	19.39	23.67	22.10
500–1000 €	35.71	37.87	37.08
1000–1500 €	17.35	16.57	16.85
1500–2000 €	6.12	7.10	6.74
2000–2500 €	10.20	4.73	6.74
2500–3000 €	3.06	1.18	1.87
>3000 €	8.16	8.88	8.61
Job Status (%)			
Employed	37.76	30.18	32.96
Unemployed	6.12	7.69	7.12
University Student	56.12	62.13	59.93

**Table 4:** Principal Component Analysis (PCA) results for the overall sample (*n* = 836).

		*H*^2^	Component				
			Healthy Behavior	Food Safety	Financially Beneficial	Media/Close Environment	Availability
Q2.8	I intend to increase my consumption of local and traditional food products.	0.574	0.748				
Q2.2	Using local and traditional food products is a good practice for my health.	0.577	0.739				
Q2.9	I want, from now on, to consume local and traditional food products.	0.616	0.733				
Q2.1	Buying local and traditional food products is a good consumer behavior.	0.556	0.720				
Q3.6	Consuming local and traditional food products is beneficial for my health.	0.561	0.696				
Q2.4	People whose opinion is important for me approve of buying and using local and traditional food products.	0.470	0.490				
Q3.3	I am afraid of jeopardizing my health by consuming local and traditional food products.	0.728		0.844			
Q3.2	Consuming local and traditional food products is hazardous for my health.	0.682		0.823			
Q3.4	Consuming local and traditional food products can cause irreversible damage to my health.	0.664		0.809			
Q2.3	It is good practice for my wage to consume local and traditional products.	0.647			0.750		
Q3.5	Consuming local and traditional food products is economically beneficial.	0.573			0.740		
Q2.5	People whose opinion is important to me recommend buying and using local and traditional food products.	0.518			0.497		
Q3.10	Media persuade me to consume local and traditional food products.	0.720				0.832	
Q3.11	Media persuade me to consume healthy products.	0.686				0.823	
Q3.12	My friends influence me to consume local and traditional products.	0.453				0.558	
Q3.9	I buy local and traditional food products from supermarkets.	0.659					0.744
Q3.7	It is hard to find local and traditional food products.	0.655					0.742
Extraction Sums of Squared Loadings (% of Variance)			24.5	13.8	9.4	6.8	6.3

*H*^2^: Communalities.

**Table 5:** PCA results for subset 1 sample (*n* = 569).

		*H*^2^	Component					
			Healthy Behavior	Food Safety	Media	Close Environment	Financially Beneficial	Availability
Q2.2	Using local and traditional food products is good practice for my health.	0.644	0.776					
Q2.8	I intend to increase consumption of local and traditional food products.	0.620	0.776					
Q2.9	I want, from now on, to consume local and traditional food products.	0.670	0.766					
Q2.1	Buying local and traditional food products is good consumer behavior.	0.636	0.730					
Q3.6	Consuming local and traditional food products is beneficial for my health.	0.606	0.677					
Q3.3	I am afraid of jeopardizing my health by consuming local and traditional food products.	0.740		0.847				
Q3.2	Consuming local and traditional food products is hazardous for my health.	0.674		0.811				
Q3.4	Consuming local and traditional food products can cause irreversible damage to my health.	0.684		0.798				
Q3.10	Media persuade me to consume local and traditional food products.	0.762			0.863			
	Media persuade me to consume healthy products.	0.731			0.845			
Q3.12	My friends influence me to consume local and traditional products.	0.477			0.552			
Q2.4	People whose opinion is important to me approve of buying and using local and traditional food products.	0.848				0.873		
Q2.5	People whose opinion is important to me recommend buying and using local and traditional food products.	0.831				0.845		
Q3.5	Consuming local and traditional food products is economically beneficial.	0.768					0.857	
Q2.3	It is good practice for my wage to consume local and traditional products.	0.727					0.800	
Q2.6	It depends on me whether or not I will consume local and traditional food products.	0.596						0.752
Q3.7	It is hard to find local and traditional food products.	0.568						0.726
Extraction Sums of Squared Loadings (% of Variance)			24.8	14.8	8.5	7.3	6.3	6.1

**Table 6:** PCA results for Subset 2 sample (*n* = 267).

		*H*^2^	Component			
			Healthy Behavior	Food Safety	Media	Availability
Q2.2	Using local and traditional food products is good practice for my health.	0.520	0.718			
Q2.9	I want, from now on, to consume local and traditional food products.	0.520	0.715			
Q2.1	Buying local and traditional food products is good consumer behavior.	0.515	0.695			
Q3.6	Consuming local and traditional food products is beneficial for my health.	0.494	0.667			
Q2.4	People whose opinion is important to me approve of buying and using local and traditional food products.	0.495	0.645			
Q2.8	I intend to increase consumption of local and traditional food products.	0.472	0.636			
Q2.5	People whose opinion is important to me recommend buying and using local and traditional food products.	0.491	0.623			
Q2.3	It is good for my wage to consume local and traditional products.	0.484	0.563			
Q3.12	My friends influence me to consume local and traditional products.	0.490	0.502			
Q3.2	Consuming local and traditional food products is hazardous for my health.	0.677		0.822		
Q3.3	I am afraid of jeopardizing my health by consuming local and traditional food products.	0.676		0.811		
Q3.4	Consuming local and traditional food products can cause irreversible damage to my health.	0.581		0.753		
Q3.11	Media persuade me to consume healthy products.	0.648			0.791	
Q3.10	Media persuade me to consume local and traditional food products.	0.672			0.777	
Q3.7	It is hard to find local and traditional food products.	0.655				−0.790
Q3.9	I buy local and traditional food products from supermarkets.	0.667				0.772
Extraction Sums of Squared Loadings (% of Variance)			25.2	13.5	8.9	8.5

**Table 7:** Linear regression of socioeconomic status with factors of two subgroups, accordingly.

Contents	Gender	Age	Education	Income
Eastern European Countries				
Healthy behavior	**	*	*	
Food Safety				
Media	*	**	**	**
Close environment				*
Financially beneficial				*
Availability				
Western European Countries				
Healthy behavior	**			**
Food Safety		*		*
Media				
Availability				**

** *p*-value < 0.01, * *p*-value < 0.05.

**Table 8:** Eastern and Western European young adults’ profile.

Eastern European Countries	Western European Countries
Good and healthy consumer behavior	Good and healthy consumer behavior
High safety standards	High safety standards
Cost (Potential high profit)	Cost
Close environment(friends, family)	Media
Availability in store	Availability in store

## Supplementary Materials

The following are available online at http://www.mdpi.com/2304-8158/7/2/22/s1.
