# Supplementary material for: What Are the Main Drivers of Young Consumers Purchasing Traditional Food Products? European Field Research
Source: Foods. 2018 Feb 12;7(2):22. doi: 10.3390/foods7020022 (PMC5848126; doi:10.3390/foods7020022)
Supplement: Supplementary file 1 [file foods-07-00022-s001.pdf]

**Supplementary material**

**University of Thessally**

Department of Agriculture Crop Production and Rural Environment
Laboratory of Agriculture Economy and Consumer Behavior

**Questionnaire**

**Part I** (about you)

1.1 Age: |___|___|
1.2 Gender: MaleFemale
1.3 Nationality:
1.4 Monthly income:
1. <500 €
2. 500–1000 €
3. 1000–1500 €
4. 1500–2000 €
5. 2000–2500 €
6. 2500–3000 €
7. >3000 €

1.5 Type of job:
1. Employed
2. Unemployed
3. University Student
4. Other (please specify)

1.6 Educational level:
1. High school graduate
2. Bachelor's degree
3. Master's degree
4. PhD's degree

**Part II** (Food) Traditional and local products are those that are produced in a specific part of a country for a very long period of time and in most cases their name is linked with the culture and the tradition of this region.
Please rate the following sentences (1–5 scale)
2.1 Buying local and traditional products is good consumer behavior.
2.2 Using local and traditional food products is good practice for my health.
2.3 It is good for my wage to consume local and traditional products.
2.4 People whose opinion is important to me approve of buying and using local and traditional products.
2.5 People whose opinion is important to me recommend that I buy and use local and traditional products.
2.6 It depends on me whether or not I will consume local and traditional products.
2.7 I don't feel good when other people see me buying local and traditional products.
2.8 I intend to increase my consumption of local and traditional products.
2.9 I want, from now on, to consume local and traditional products.

**Part III** Please rate the following sentences (1–5 scale)
3.1 Health is better than wealth.
3.2 Consuming local and traditional products is dangerous for my health.
3.3 I am afraid of jeopardizing my health by consuming local and traditional food products.
3.4 Consuming local and traditional products can cause irreversible damage to my health.
3.5 Consuming local and traditional products is economically beneficial.

3.6 Consuming local and traditional products is beneficial for my health.
3.7 It is hard to find local and traditional products.
3.8 I buy local and traditional food products from small local shops.
3.9 I buy local and traditional food products from supermarkets.
3.10 Media persuade me to consume local and traditional products.
3.11 Media persuade me to consume healthy products.
3.12 My friends influence me to consume local and traditional products.
